# Gut microbiota and irritable bowel syndrome: status and prospect

**DOI:** 10.3389/fmed.2024.1429133

**Published:** 2024-10-17

**Authors:** Xinyu Cheng, Cheng Ren, Xiaofei Mei, Yufeng Jiang, Yafeng Zhou

**Affiliations:** ^1^Department of Cardiology, The Fourth Affiliated Hospital of Soochow University, Medical Center of Soochow University, Suzhou Dushu Lake Hospital, Suzhou, China; ^2^Department of Cardiology, The First people’s Hospital of Zhangjiagang, Affiliated Hospital of Soochow University, Medical Center of Soochow University, Zhangjiagang, Jiangsu, China; ^3^Institute for Hypertension, Soochow University, Suzhou, China

**Keywords:** irritable bowel syndrome, gut microbiota, gut-brain axis, fecal microbiota transplantation, brain-gut-microbiome

## Abstract

Irritable bowel syndrome (IBS) is a very common gastrointestinal disease that, although not as aggressive as tumors, affects patients’ quality of life in different ways. The cause of IBS is still unclear, but more and more studies have shown that the characteristics of the gut microbiota, such as diversity, abundance, and composition, are altered in patients with IBS, compared to the healthy population, which confirms that the gut microbiota plays a crucial role in the development of IBS. This paper aims to identify the commonalities by reviewing a large body of literature. Changes in the characteristics of gut microbiota in patients with different types of IBS are discussed, relevant mechanisms are described, and the treatment modalities of gut microbiota in IBS are summarized. Although there are more clinical trials that have made good progress, more standardized, more generalized, larger-scale, multi-omics clinical studies are what is missing. Overall, gut microbiota plays a crucial role in the development of IBS, and there is even more potential for treating IBS by modulating gut microbiota.

## Introduction

Irritable bowel syndrome (IBS) is a common chronic gastrointestinal functional disorder characterized by persistent or intermittent abdominal pain, bloating, and changes in bowel habits, along with abnormal stool characteristics (1). According to the Rome IV, IBS can be classified into four subtypes: constipation-predominant (IBS-C), diarrhea-predominant (IBS-D), mixed (IBS-M), and unsubtyped (IBS-U) (2). Although IBS has no identifiable organic pathology in the intestines, it adversely affects the quality of life and work productivity of patients and also can lead to disability (3). Epidemiological studies show that the global prevalence of IBS is about 10–20% and continues to increase yearly (4). Currently, IBS lacks definitive diagnostic criteria and is clinically diagnosed based on the patient’s symptoms and medical history, alongside imaging and endoscopy to exclude other organic diseases (5). The exact pathophysiology of IBS remains unclear, but it may be related to changes in gastrointestinal motility, visceral hypersensitivity, low-grade mucosal inflammation, environmental factors, gut microbiota imbalance, and psychosocial disorders (6).

**Figure 1:**
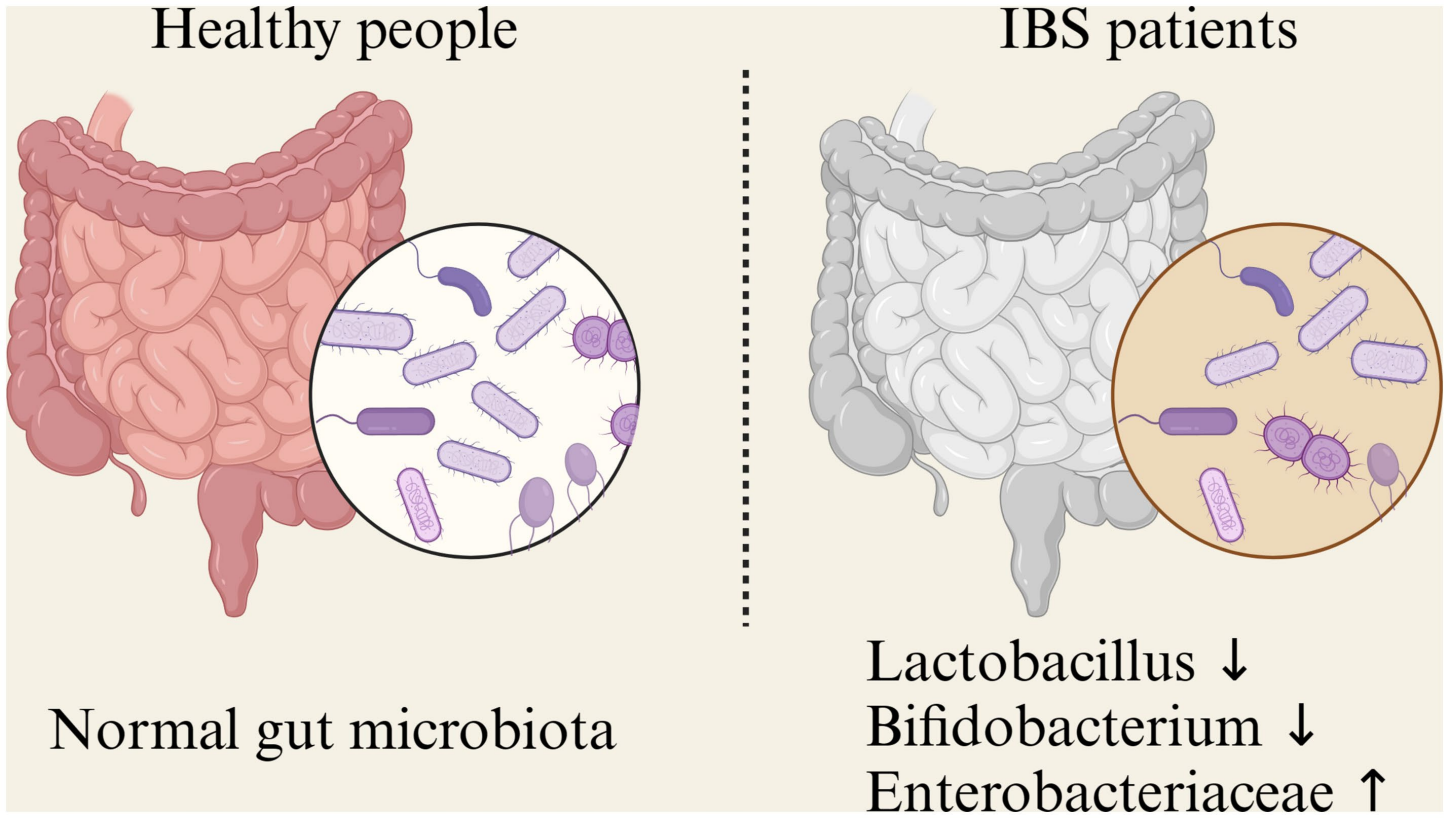
Main microflora alterations in irritable bowel syndrome (IBS).

The gut microbiome is a collective term for the complex ecosystem of microorganisms in the intestines, including bacteria, viruses, fungi, and protozoa. Bacteria dominate, constituting over 99%, thus it is also known as the gut microbiota. High-throughput sequencing reveals that the human gut contains about 500–1,000 species of microbes, with a count of up to 10^14^, approximately 10 times the number of human cells (7). In healthy adults, over 90% of gut bacteria belong to four dominant phyla: *Firmicutes*, *Bacteroidetes*, *Actinobacteria*, and *Proteobacteria*. Other phyla have much lower abundances (8). Based on their functions, intestinal microbiota can be broadly classified into two categories:functional bacteria and pathogenic bacteria. Functional bacteria primarily including *Bacteroides*, *Clostridium*, *Bifidobacterium*, and *Lactobacillus*. They are abundant, comprising over 99% of the gut microbiota. Commensal bacteria can synthesize various vitamins, use protein residue to produce essential amino acids, and participate in carbohydrate and protein metabolism. They also facilitate the absorption of minerals like iron, magnesium, and zinc. They also contribute to food digestion, stimulate gut motility, inhibit pathogenic bacteria growth, and break down harmful and toxic substances. The second type is the pathogenic bacteria, including opportunistic pathogenic bacteria and pathogenic bacteria. Opportunistic pathogenic bacteria, primarily *Enterococcus* and *Enterobacteriaceae*. When the immune system is weakened, these bacteria can multiply excessively or migrate, leading to gut environment imbalance and intestinal disease. Although not numerous, they are highly mobile, considered foreign bacteria, and represent unstable factors in the gut. Pathogenic bacteria, primarily *Salmonella* and pathogenic *Escherichia coli*. They usually enter the gut through accidental ingestion and produce harmful substances like nitroso compounds and colicins, affecting immune function and causing various diseases (9). When the body is influenced by environmental factors, diet, medication, and other external elements, the composition and function of the gut microbiota can change, a condition known as gut dysbiosis (Figure 1).

Notably, current gut microbiota samples are usually collected via stool samples, which is easy to implement in clinical practice but has significant limitations. Some studies suggest that the mucosa-associated microbiota is more closely linked to host cells (10). One study used ingestible devices to collect samples from different regions of the small intestine and compared them to stool samples, revealing significant differences in microbiome composition. Li et al., through 16S rRNA sequencing of duodenal, rectal, and fecal samples, found that the alpha diversity and phylogenetic diversity of the duodenal microbiota were higher than those of the rectal and fecal samples. Their study also revealed that the duodenal samples had a higher abundance of *Proteobacteria*, with *Acinetobacter* and *Prevotella* being predominant in the duodenum, while *Bacteroides* and *Prevotella* dominated in the rectum (11). This offers new insights for future research on gut microbiota.

## Microbial dysbiosis in IBS subtypes

So far, increasing evidence suggests that microbial dysbiosis leads to the development of IBS (12). This results from disruptions in host-microbiome interactions (13). By leveraging increasingly advanced next-generation sequencing technologies, such as metagenomics and 16S rRNA sequencing, our understanding of microbial ecology and host–microbe interactions has now reached the DNA level (14). High-throughput sequencing has revealed changes in microbial diversity, abundance, and composition in IBS.

**Figure 2:**
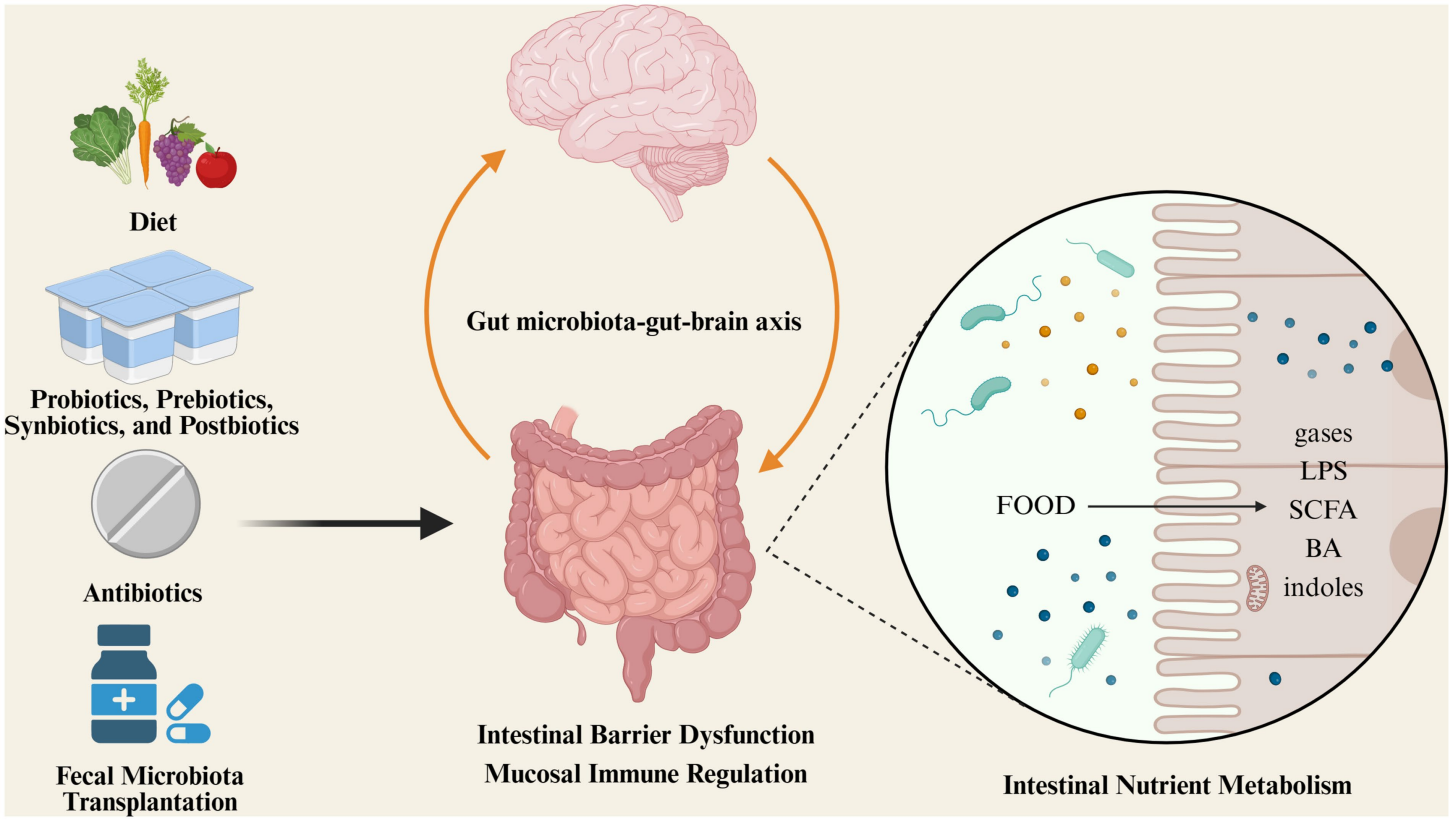
Gut microbiota influences the progression of IBS through different mechanisms; microbiota-targeted therapeutic approaches in IBS.

At the phylum level, most studies show that *Proteobacteria* increase in IBS patients (15). Several studies have also shown that IBS patients have a reduced number of *Lactobacillus species*, while the abundance of *Ruminococcus gnavus* and *Lachnospiraceae* is significantly higher. In contrast, *Barnesiella intestinihominis* and *Coprococcus catus* are found in notably lower abundance in IBS patients (16–18). Although some research indicates no significant differences between IBS patients and controls (12). Research on the other three phyla (*Bacteroidetes*, *Actinobacteria*, and *Firmicutes*) shows varying results (Figure 2).

Some consistent findings exist: Jeffery’s observations show that IBS-D patients have reduced levels of *Lactobacillus* and *Bifidobacterium*, with elevated *Enterobacteriaceae* levels compared to healthy controls (16). Wang’s meta-analysis shows that probiotic levels decrease while pathogenic bacteria levels increase in IBS-D patients, This finding may reflect the patients’ history of prior gut infections. Another possible explanation for this observation could be changes in the gut environment, including a reduction in strict anaerobes, as well as increased inflammation and gastrointestinal motility, which favor the growth of facultative and less fastidious bacteria, such as *Enterobacteriaceae*. However, there may be some bias in these data (19). A meta-analysis of a randomized controlled trial studying the gut microbiota of IBS-C patients shows a significant increase in fecal Bacteroides, with no notable increase in *Bifidobacterium*, *Lactobacillus*, *Enterobacteriaceae*, or Enterococcus colony counts, and no differences in gut colonization resistance compared to healthy individuals (20). Research findings on IBS-M patients vary widely, A study by Soldi et al. found that the levels of *Faecalibacterium prausnitzii* were reduced in IBS-M patients, but increased after treatment with rifaximin, suggesting that *F. prausnitzii* may have some specificity in IBS-M. However, given the small sample size, the generalizability of these findings remains uncertain. But all further confirm that the development of this condition is closely tied to gut dysbiosis. Due to the small proportion of IBS-U patients, research focusing on this subtype is limited. A study on a South Indian population found an increase in *Pseudomonas aeruginosa* in IBS-U patients (21).

Differences in experimental methodology, target populations, psychological stress, and dietary habits lead to inconsistencies in microbial diversity and abundance among IBS patients (22). To better understand the relationship between the gut microbiota and IBS, experimental processes, such as inclusion criteria, specimen processing workflows, and data analysis, should be standardized for more reliable results. Furthermore, longitudinal and integrated multi-omics analyses are necessary for a deeper understanding of the gut microbiome’s role (14).

## The role of microbial dysbiosis in IBS pathogenesis

Based on the aforementioned studies, changes in the gut microbiota are related to the development and progression of IBS. However, the causal relationship is still unclear. Physiological changes in the gut caused by IBS could be responsible for changes in the gut microbiota and its functions. Conversely, gut dysbiosis may be the key initial factor for pathophysiological changes in IBS (23). Current evidence indicates that these conditions may coexist. In any case, the gut microbiota’s effects on nutrient metabolism, gut barrier dysfunction, mucosal immunity, and the brain-gut-microbiome (BGM) axis are crucial for understanding why it plays a vital role in the onset and persistence of IBS.

### Intestinal nutrient metabolism

The metabolic substrates of the gut microbiota mainly come from undigested or indigestible food and endogenous mucus secreted by intestinal epithelial cells. Through a series of metabolic processes, gut bacteria produce beneficial and harmful metabolites, such as gasses (hydrogen sulfide, methane), lipopolysaccharides, peptidoglycans, short-chain fatty acids (SCFAs), bile acids, and indoles.

Previous studies have shown that the gut microbiota of IBS-C patients exhibit greater distinctiveness and variability compared to healthy individuals and IBS-D patients (14). Among the various bacterial metabolic products, short-chain fatty acids (SCFAs) are globally acknowledged for their crucial role in maintaining colonic homeostasis (24). SCFAs are the by-products of carbohydrate fermentation by symbiotic anaerobic bacteria, playing a key role in preserving gut barrier integrity, regulating immune function, and possessing anti-inflammatory properties. Studies have shown that the levels of short-chain fatty acids are reduced in IBS-C patients compared to those with IBS-U and IBS-D (25). Therefore, short-chain fatty acids have the potential to serve as biomarkers for IBS. Additionally, evidence suggests that lipopolysaccharides, derived from commensal bacteria, regulate IBS-related visceral sensation, mucosal inflammation, and gut barrier dysfunction by activating toll-like receptor 4 (TLR4) (26). Changes in the abundance and diversity of the gut microbiota also have a profound impact on bile acid metabolism, ultimately leading to IBS (27). In summary, gut microbiota metabolites may act as messengers, regulating host gastrointestinal function and influencing the occurrence and progression of IBS.

### Intestinal barrier dysfunction

In one study, pre-treatment with a *Lactobacillus rhamnosus* derivative prevented increased intestinal permeability in IBS patients, alleviating symptoms. This indicates a link between gut dysbiosis and intestinal barrier function in IBS (28). Changes in the gut microbiota could impair intestinal barrier function in IBS patients through various mechanisms, including metabolic and immune pathways. Previous studies have shown that increased intestinal mucosal permeability in IBS patients is likely related to the expression and distribution of tight junction protein ZO-2 in the intestinal epithelium (29). *E. coli* promotes ZO-2 expression, enhances the barrier function of epithelial cell tight junction complexes, and can even repair the intestinal epithelial barrier damaged by pathogenic *E. coli* (30). Additionally, in IBS patients, the expression levels of tight junction proteins such as Occludin and Claudin-1 were found to be reduced in the duodenum, jejunum, and colon (31, 32). Interestingly, the microbiota has been found to regulate the expression of these tight junction proteins (33), and an increased passage of bacteria through the intestinal barrier has been observed (34). This further demonstrates that gut dysbiosis contributes to the development of IBS by causing intestinal barrier dysfunction.

### Mucosal immune regulation

Several studies have shown that the mucosal immune system in IBS patients is imbalanced (35), characterized by elevated inflammatory factor expression and immune cell activation. It has been reported that in IBS-D patients, the levels of 5-HT and 5-HT3 receptors in the intestinal mucosa are significantly higher than in healthy controls, indicating that the 5-HT system is impaired in IBS patients (36). An interesting finding in IBS patients is the increased expression of Toll-like receptors (TLRs) (37). These receptors are present in various cells, including intestinal epithelial cells and immune cells, and are closely related to neural and immune receptors involved in regulating intestinal mucosal homeostasis (38). TLRs recognize specific microbial components of both commensal and pathogenic bacteria, playing a role in immune tolerance to commensals and defense against pathogens (39). However, the exact pathways and targets by which the gut microbiota affects intestinal immunity are still debated and remain inconclusive.

### Gut microbiota-gut-brain axis

The brain-gut axis is a bidirectional neural pathway consisting of the central nervous system, the enteric nervous system, and the autonomic nervous system, including visceral sensory conduction, neuroendocrine-immune regulation, and stress response pathways (40). Some researchers believe the brain-gut axis plays a key role in IBS pathogenesis, and the gut microbiota may also participate in this process. The interaction between the gut microbiota and the gut-brain axis is called the gut microbiota-gut-brain axis (41). Research indicates that the vagus nerve is a primary regulator of the microbiota-gut-brain (MGB) axis. It is composed of somatic and afferent fibers (80%) and general and special visceral efferent fibers (20%). Under normal conditions, the vagus nerve has sensory functions and is activated by diet-responsive gut microbes, metabolites, endocrine factors, enzymes, and neurotransmitters (42). Each of these factors can be influenced by changes in the microbiota composition and play a role in IBS pathology. In the gut, the vagal nerve endings synapse onto neurons in the enteric nervous system (ENS), which controls the function of gastrointestinal muscles, neurohormones, and secretion systems to produce functional digestive patterns. In IBS, the pathophysiology involves alterations in the gut microbiota composition, compromised mucosal integrity, and low-grade inflammation (6). Beyond the circulatory pathways, some of these factors may also trigger fluctuations in ENS activity, thereby impacting the brain. This bidirectional signaling relationship can be disrupted by chronic IBS. In the brain of chronic IBS patients, efferent signals may be perceived as unpleasant or painful, potentially leading to chronic visceral discomfort or pain (43). Gut dysbiosis activates the intestinal mucosal immune system, thereby disrupting the intestinal epithelial barrier function and causing visceral hypersensitivity and gastrointestinal motility disorders in IBS patients, which leads to abdominal pain, diarrhea, or worsening of existing symptoms. The gut microbiota and the brain are interconnected and can influence each other, jointly regulating gastrointestinal neural, endocrine, and immune functions, thus playing a role in the development and progression of IBS.

## Microbiota therapeutic approaches in IBS

Increasing evidence indicates that changes in the gut microbiota are related to the development and progression of IBS. Regarding current gut microbiota treatment strategies, many promising results have been achieved, such as gut microbiota-targeted therapy being recommended for diarrhea-predominant IBS (IBS-D). Currently, finding safer and more effective gut microbiota-based treatments has become a major focus of IBS research.

### Diet

In recent decades, dietary control has gradually become more prominent in IBS treatment. Initially, dietary recommendations for IBS were drawn from guidelines by the National Institute for Health and Care Excellence (NICE) (44) and the British Dietetic Association (BDA) (45). They emphasized self-management through lifestyle, diet, exercise, and symptom-based medication education. Recent studies show that a low FODMAP (fermentable oligosaccharides, disaccharides, monosaccharides, and polyols) diet has significant advantages over the BDA/NICE diet in alleviating certain clinical symptoms, especially bloating (46). The FODMAP diet increases water in the colon due to osmotic effects, leading to diarrhea. It may also be fermented by the gut microbiota, resulting in excessive gas that worsens bloating in IBS patients. However, a low FODMAP diet reduces fiber intake and may cause constipation in some patients (47). Therefore, IBS patients need to adjust according to their individual conditions.

Studies have found that a low FODMAP diet significantly reduces Bifidobacterium levels and decreases the overall bacterial count, thereby changing the gut microbiome composition (48). A comprehensive systematic review and meta-analysis of 12 trials involving 772 patients studied the effects of a low FODMAP diet on IBS symptoms in most studies. Results showed a significant improvement in IBS severity according to the IBS Severity Scoring System (IBS-SSS), with higher IBS-QoL scores compared to the control group (49). However, a low FODMAP diet does not have the same effect on different IBS subtypes, which may be related to the specific components of the diet (Table 1).

**Table 1:** Differences in gut microbiota among IBS subtypes across various studies.

Variable	Different bacterial populations	Country	References
IBS-C	*Lactobacillus* (−), *Bifidobacterium* (−) *Bacteroides thetaiotamicron* (+), *P. aeruginosa* (+), Gram-negative bacteria (+), *Veillonella* (+)	France, Netherlands, China, Spain, Finland, Japan Northern India	Wang et al. (19) Shukla et al. (20)
IBS-D	*Lactobacillus* (−), *Bifidobacterium* (−), *Enterobacteriaceae* (+) *Lactobacillus (−)*, *Bifidobacterium* (−), *E. coli* (+) *Bacteroides thetaiotamicron* (+), *P. aeruginosa* (+), Gram-negative bacteria (+)	Ireland United States, Netherlands, China, Spain Northern India	Jeffery et al. (16) Wang et al. (19) Shukla et al. (20)
IBS-M	*Faecalibacterium prausnitzii* (−)	Italy	Soldi et al. (63)
IBS-U	*P. aeruginosa* (+)	Northern India	Shukla et al. (20)

### Probiotics, prebiotics, synbiotics, and postbiotics

Probiotics are orally active microorganisms that can colonize the gastrointestinal tract (50). Prebiotics are indigestible food components that selectively promote the growth or activity of beneficial bacteria to improve patient symptoms (51). Synbiotics are mixtures of probiotics and prebiotics (52), while postbiotics are active substances produced by bacteria, such as bacteriocins, vitamins, and short-chain fatty acids (SCFAs) (53). Several randomized controlled trials (RCTs) show that treatment with specific probiotics, such as *Lactobacillus plantarum*, *Bacillus coagulans*, or multi-strain probiotic formulations, significantly improves abdominal pain, bowel movement frequency, and quality of life in IBS-D patients (54). A meta-analysis of 10 studies involving 757 patients evaluated the effects of probiotics on IBS-C patients. Compared to a placebo, probiotics significantly improved diarrhea symptoms but did not show significant differences in abdominal pain or bloating (55). Studies on prebiotics found that taking non-inulin fructooligosaccharides significantly improved gastrointestinal bloating (56), but research on synbiotics and postbiotics remains multiple.

Although the American Gastroenterological Association currently does not support probiotics for IBS treatment (57), it cannot be denied that they could be a breakthrough treatment. However, this requires further large-scale RCTs to clarify the role of these compounds in treating IBS.

### Antibiotics

Research indicates that using antibiotics to reset the gut microbiota could be a novel approach for treating IBS. Several randomized controlled trials show that antibiotics like rifaximin, neomycin, and norfloxacin can alleviate IBS symptoms (58). However, indiscriminate use of antibiotics can lead to side effects, adverse reactions, antibiotic resistance, and severe gut dysbiosis, limiting their application in IBS treatment (59). Currently, rifaximin is the only antibiotic approved by the U.S. Food and Drug Administration (FDA) for IBS treatment, but its approved application range is very limited (57). The standard dosage is 550 mg, taken three times daily for 2 weeks.

### Fecal microbiota transplantation

With increasing awareness of the relationship between gut dysbiosis and disease onset, FMT has gained more attention in the past decade. FMT was first approved by the FDA in 2013 for the treatment of *Clostridium difficile* infection (60). Since then, multiple studies have explored FMT for IBS patients, but results have shown inconsistencies. Although there is significant controversy regarding the effectiveness of FMT for IBS, some studies indicate that IBS patients can benefit from it (61). A systematic review summarized results from seven randomized controlled trials (RCTs) on FMT for IBS, and four RCTs found that FMT was effective for IBS outcomes, including symptom relief and improved quality of life, while the other three showed no effect (62). However, the administration methods and adverse reactions of FMT remain significant concerns. Currently, FMT is not recommended as a primary treatment option for IBS patients, as there is still a long way to go in exploring this approach.

## Reflections and perspectives

IBS is a common multifactorial functional gastrointestinal disease. Despite its benign nature, it remains challenging to manage and treat fully.

Research over the past few decades shows that gut dysbiosis is closely linked to the development and progression of IBS, though the causal relationship remains unclear. Nevertheless, the effects of gut dysbiosis on intestinal nutrient metabolism impairment, intestinal barrier dysfunction, mucosal immunity, and the brain-gut-microbiome (BGM) axis are key factors influencing the development and progression of IBS. Identifying the precise composition of the gut microbiota is crucial to unraveling this relationship and unlocking the mysteries of IBS.

Although treatment methods targeting the gut microbiota are currently contentious, they still offer promising prospects for IBS patients. This paper aims to analyze the relationship between the gut microbiota and IBS, as well as potential treatment options. Since every patient’s gut microbiota composition differs, individual responses to the same treatment can vary. Therefore, new detection methods and stable treatment approaches need in-depth exploration, such as whether the mucosa-associated microbiota aligns with the fecal microbiota, and how prebiotics, synbiotics, postbiotics, and fecal transplantation can be optimized for breakthroughs in the coming years.

Multi-omics analysis is enabling a transition toward personalized IBS characterization and treatment plans. However, this requires further longitudinal multi-omics studies and functional research into the genetic basis and mechanisms underlying gut microbiota-host interactions. Such research will enhance our understanding of the role of gut colonizers in IBS, guiding the development of more targeted dietary and therapeutic approaches, and ultimately leading to microbiome-targeted therapies.
